# Learning better by repetition or variation? Is transfer at odds with task specific training?

**DOI:** 10.1371/journal.pone.0174214

**Published:** 2017-03-23

**Authors:** Emmanuel Bonney, Lemke Dorothee Jelsma, Gillian D. Ferguson, Bouwien C. M. Smits-Engelsman

**Affiliations:** 1 Department of Health and Rehabilitation Sciences, University of Cape Town, Cape Town, South Africa; 2 Department of Physical Therapy, University of Ghana, Accra, Ghana; 3 Developmental and Clinical Neuropsychology, University of Groningen, Groningen, the Netherlands; SUNY Downstate MC, UNITED STATES

## Abstract

**Objective:**

Transfer of motor skills is the ultimate goal of motor training in rehabilitation practice. In children with Developmental Coordination Disorder (DCD), very little is known about how skills are transferred from training situations to real life contexts. In this study we examined the influence of two types of practice on transfer of motor skills acquired in a virtual reality (VR) environment.

**Method:**

One hundred and eleven children with DCD and their typically developing (TD) peers, aged 6–10 years (M = 8.0 SD = 1.0) were randomly assigned to either variable (n = 56) or repetitive practice (n = 55). Participants in the repetitive practice played the same exergame (ski slalom) twice weekly for 20 minutes, over a period of 5 weeks, while those in the variable group played 10 different games. Motor skills such as balance tasks (hopping), running and agility tasks, ball skills and functional activities were evaluated before and after 5 weeks of training.

**Results:**

ANOVA repeated measures indicated that both DCD and TD children demonstrated transfer effects to real life skills with identical and non-identical elements at exactly the same rate, irrespective of the type of practice they were assigned to.

**Conclusion:**

Based on these findings, we conclude that motor skills acquired in the VR environment, transfers to real world contexts in similar proportions for both TD and DCD children. The type of practice adopted does not seem to influence children’s ability to transfer skills acquired in an exergame to life situations but the number of identical elements does.

## Introduction

Active video games, also known as exergames have recently been found to be useful tools for training children with and without Developmental Coordination Disorder (DCD) [1–5]. For instance, the Nintendo Wii has been shown to improve dynamic balance, aerobic capacity and agility in children with motor problems [6]. Exergames are motion-dependent video games that use whole bodily movement to regulate gameplay [7]. Exergames provide online feedback and motivational experiences making it conducive for children with DCD to learn new skills. For exergames to be relevant and effective as an intervention, it is critical to demonstrate that skills learned by playing active video games can transfer to motor skills used in the real world.

Motor learning researchers have indicated that the best way to enhance transfer and retention is to train learners using a variable practice structure rather than repetitive practice [8]. Although repetitive practice leads to better performance during the acquisition phase of training, it often impairs learners’ ability to retain and transfer skills acquired in practice to other situations. Variable practice on the other hand, results in greater retention and transfer because it strengthens generalized motor programs and creates memory representations enabling individuals to achieve desired movement proficiency even in unaccustomed settings [9].

Transfer of learning is the ability to apply acquired motor skills to novel task variants [10,11]. From this definition, it is clear that the core element of transfer of skills is generalizability or adaptability. Generally, transfer is described in several ways including near vs. far transfer [12], positive and negative transfer [13] and bilateral transfer, showing improvement of a task not only in the trained limb, but also in the non-trained hand or foot [14]. While near transfer reflects transfer effects that occur between similar skills and/or tasks, far transfer is usually used to denote transfer effects observed between two dissimilar skills (i.e. tasks that share fewer common elements) [15]. Critically, far transfer is the application of learned skills in unfamiliar contexts.

Two theoretical paradigms that are commonly cited to explain the idea of skill transfer are the identical elements theory and the transfer appropriate processing theory [16]. According to the identical elements theorists, the degree of transfer depends on the level of similarity (number of common elements) between two skills. Thus, the greater the number of common elements, the stronger the transfer effects. In contrast, proponents of transfer-appropriate processing theory argue that transfer effects depend on the similarity of cognitive processing features between skill sets and not so much on how motor elements are related [17].

The concept of transfer of training has been extensively explored in the motor learning literature. However, most studies have almost exclusively focused on transfer effects occurring within natural or real-world settings in healthy individuals [18,19]. To date, the number of studies investigating transfer from virtual environments to the physical world is limited. Additionally, there is no evidence of how children with and without DCD transfer motor skills acquired from practice situations to untrained skills outside training contexts. It is therefore reasonable to investigate whether motor skill training delivered in a virtual environment would lead to improvement in skills in real world settings. Even though recent research has demonstrated the occurrence of skill transfer in children with DCD [20], the exact nature of the transfer mechanism is not known.

DCD is one of the most common motor disorders of childhood and is considered to be a global health problem among school-aged children [21]. The prevalence of DCD is estimated to be between 5 to 6% worldwide [22], These limitations may affect their academic performance and social life [23]. Normally, children must be proficient in a wide array of motor skills to be able to function effectively and interact in their environment. Unfortunately, for those with DCD, time and resource constraints do not allow them to receive intervention for every essential motor skill. The ultimate goal for prescribing intervention for children with DCD is to equip them with functional skills required during daily activities, leisure or sports. It is therefore expedient to gain more insight into the mechanisms of skill transfer in this population, so clinicians could offer training programs in ways that would enhance generalizability of skills.

It is now known that the most effective interventions for treating children with DCD are task-specific approaches [1,24]. Commonly reported task-oriented approaches found to be effective treatment options for children with DCD include Cognitive Orientation to Occupational Performance Approach (CO-OP) [25,26], Neuromotor Task Training (NTT) [27–31] and Virtual Reality gaming [2,3,32].

It is widely reported that children experience greater gains in motor performance when children are active participants in therapy, with limited therapist support, rather than therapist-directed interventions, where children are given high doses of external support [33]. Motor learning is enhanced when children are presented with many options and are given opportunities to make choices [34,35]. In addition, increasing the number of practice trials and time on task further increases the extent of motor learning [36,37]. Based on these considerations, we designed the present study to investigate the impact of type of practice (variable vs. repetitive) on a wide range of transfer tasks in two groups of children who were trained to acquire dynamic balance skills using an active video game (Nintendo Wii).

Previous virtual reality (VR) training using the Nintendo Wii revealed positive transfer effects on balance and agility tasks in children who played active video games with variable practice schedule [2,3,7]. Studies conducted in typically developing (TD) children and other clinical groups have shown that positive transfer occurs when trained skills are similar to the new task, so called near-transfer [13] as well as to tasks that are dissimilar (far transfer) [38,39]. Yet, it is not clear whether variable practice would lead to positive far transfer in children with and without DCD. Therefore, the purpose of the study was to test whether VR training leads to improvements in every day skills requiring dynamic balance such as hopping, to tasks with some common motor elements, such as slalom running and sit-to-stand. Lastly, we also included tasks with very few identical elements to the training (ball catching, pegboard). We compared a VR protocol of variable practice with repetitive practice and examined the changes on transfer between TD and DCD children. We hypothesized that children who played active video games and followed the variable practice protocol would demonstrate greater gains in skill transfer than the repetitive practice group.

## Methods

### Overview of research design

A stratified randomized pre-post single blinded design was used to evaluate practice effects on transfer of motor skills using the Nintendo Wii program in children with and without DCD.

### Participants

#### Identification of DCD and controls

In this study, participants were selected using the same procedure described in earlier studies [40,41]. Teachers and parents were asked to assist in identifying children with motor coordination problems based on their observation of the children in class and on the playground. The four *DSM-5* criteria were then used to identify children with DCD [22]. All children in the age range of 6–10 years (Criterion C), who scored below the 5^th^ percentile on the Movement Assessment Battery for Children 2^nd^ edition (Criterion A), who were identified as having a motor coordination problem by the teacher or parent (Criterion B), whose parents reported no diagnosis of a significant medical condition or co-morbidity known to affect motor performance in the parental questionnaire (Criterion D); and whose teacher affirmed the absence of intellectual or cognitive impairment (Criterion D) appeared to fulfill the criteria for DCD. Through this procedure, 57 children with DCD were selected to take part in the study and they were age- and gender matched with 54 TD children from the same classes.

TD children recruited from the same school as the children with DCD had: 1) no evidence of functional motor problems as observed by their teacher or parent, 2) a score above the 15^th^ percentile on the MABC-2, 3) no diagnosis of a significant medical condition as reported by a parent and 4) absence of intellectual or cognitive impairment as confirmed by their parent and teacher. None of the children that took part in the study had experience or owned commercially available consoles for active computer games.

### Instruments

#### The Movement Assessment Battery for Children-2 (MABC-2)

The MABC-2 [42] consists of eight physical items of three subtests used to assess motor coordination in children aged 3–16 years. Three age bands (3–6; 7–10; 11–16 years) offer age appropriate items. Raw scores for each item are converted into standard scores. The Total Standard Score (TSS) is a sum of the individual standard scores and gives an impression of overall motor proficiency. The MABC-2 is considered a reliable and valid measure for the assessment of motor performance [42–44]. In children with DCD, internal consistency is reported to be high (alpha = 0.90) and test-retest reliability for the total scores is viewed as excellent (ICC = 0.97). The age appropriate MABC-2 items were used to confirm the motor performance of both participant groups and at post test to determine the change in overall motor proficiency s after playing exergames.

It is known that the MABC-2 balance items have a ceiling effect in TD children. Since all children in the current study were between 7–10 years (age band 2), we also used the items of age band 3 as additional standardized balance items. Scores of these three items were added up and used for pre and post training comparison to have a broad range of balance items for evaluation of the transfer effect. We refer to these items in the text as Balance AB3.

#### Running and agility and balance measures (Bruininks Oseretsky test of motor proficiency 2, BOT2) [45]

Based on earlier research [2,46], we expected the largest transfer of exergaming to activities that require static and dynamic balance, and fast directional changes. For that reason, we chose two subtests of the Bruininks Oseretsky test of Motor Proficiency, second edition [45] to measure balance and agility. First, the balance subtest which consists of seven static balance tasks and two dynamic balance tasks. Secondly, the running speed and agility subtest, which consists of one sprint task and four dynamic balance tasks. Each raw score is converted into a point score. All point scores are cumulated into a total point score for each subtest. Per subtest, total point scores are converted according to sex- and age specific norm tables into subtest scale scores, which were used for the analysis. Inter-rater reliability for scale scores is consistently high for subtest balance (0.99), and running speed & agility (0.99) [45].

#### Functional strength tasks

To test if improvements in balance could be extrapolated beyond balance activities and be applied in standardized tasks that are closely related to every day motor skills, we chose activities from the Functional Strength Measure (FSM) [47]. These items are standardized and have age appropriate norms. For this study, the 5 items of the FSM were administered; the Long jump. Lateral step-up, Sit to stand, Stair climbing and Lifting a box [47,48]. Each test item was administered three times and the best raw scores were used for the analysis. Reliability of the FSM is reported to be good with ICC’s for the various items ranging from, 0.73 to 0.91 [48].

#### Sprinting

A frequent playground activity commonly observed in most primary schools is playing tag games and for that children need to run short distances and sharply change direction. To get an impression of the speed at which children could run and turn over short distances we chose to administer two sprint tests. In the 10 x 5-meter sprint test, children have to run a distance of 5m ten times without stopping [49,50]. After every 5m the child has to turn. The time to complete the 10 laps (measured in seconds) is recorded. The test has good reliability in TD children [49,50]. No test retest data are available for DCD children yet.

**Fig 1:**
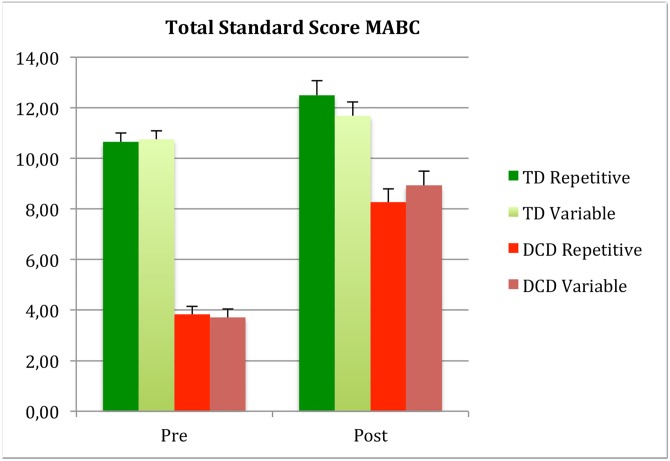
Depicting MABC TSS scores pre and post training for participant groups and protocol.

To increase the agility component, a new test item was developed: the 10 x 5-meter slalom test [7]. It is similar to the 10 x 5m sprint test, except that the trajectory that the children have to run requires multiple directional changes to be made during the test. Children had to complete ten laps and the time was recorded (See Fig 1).

### Training

In a special classroom at the school, a fixed arrangement of four televisions, four Nintendo Wii Fit consoles and balance boards were set up and partitioned in a way that children could not see other screens while playing. Four children simultaneously played the game under the direct supervision of two trained (student) therapists, who provided instructions when needed, motivation and encouragement to the children. These student therapists also recorded scores obtained by each child at the end each game. All children spent 20 minutes playing twice a week for a period of five weeks. Both groups were randomly divided in two training protocols:

The *variable practice protocol*: children could play several games in random order choosing their preferred games out of 10 preselected Wii Fit balance games. For details about the games see S1 Appendix. They were allowed to play selected games at least once per session.

The *repetitive practice protocol*: children could only play the ski slalom game in alternating series of beginners (19 gates) and advanced level (27 gates).

Children were offered an opportunity to catch up if they missed a training session, preferably during the same or otherwise the next week.

### Procedure

Approval for the study was granted by the University of Cape Town, Faculty of Health Sciences Human Research Ethics committee (HREC: 556/2015) and the designated educational authorities. Written informed consent for the testing was obtained from all parents and written informed assent was also given by each child.

Children with DCD were tested by a team of qualified physiotherapists and physiotherapy students who had received prior training on the administration of all outcome measures before commencement of the study. To ensure that the protocols both contained approximately equal numbers of children with DCD and TD, all participants were stratified by participant group (TD/DCD) before being randomly assigned to either protocol.

Children were tested on the FSM items and BOT-2 by the same assessors on one day and on 10 x 5 meter sprint tests on a separate day. Training commenced one week later and continued for five weeks. All pre- and post-measures were conducted by two separate team of assessors, who were blinded to pretest results and the protocol the children were assigned to.

### Data analysis

All data were checked for normality and equality of variances and appropriate analyses are reported. Differences in demographic characteristics between the groups were calculated at baseline using Pearson’s Chi squared test (gender and handedness) and t-test for age, height, weight, and BMI, and pretest values of the tests (motor performance, balance, agility and strength).

Next we tested if motor performance, balance, agility and the functional strength tasks changed with training and if this effect was different between the protocols and participant groups, by using a repeated measure ANOVA with pre and post score as within, and protocol (repetitive/variable) and participant group (TD/DCD) as between factors.

Lastly, post hoc paired t-tests were done to compare pre-and post values for the TD and DCD group separately.

Effect sizes were reported so the magnitude of the effects is easily understood regardless of the scale that was used to measure the dependent variable. This would also allow for comparison to be made between the results of the current study and those that have been reported in the literature. The size of the between group effects was determined by Cohen’s d effect size and classified as: >.20 small, >.50 moderate and >.80 large effect size [51]. For the ANOVA, the estimates of effect size were calculated as partial eta square. Partial eta squared is to be interpreted as the proportion of the total variability in the outcome variable that is accounted for by the variation in the independent or manipulated variable (in our case intervention and protocol). Small, medium, and large effects correspond to values of η^2^ of 0.01–0.05, 0.06–0.14 and >0.14 [52].

Significance level was set at *p* < .05. All statistical analyses were run in Statistical Package for the Social Sciences (SPSS Inc., version 23).

The total sample size required in each group was calculated based on previous data [31]. Accordingly, it was established that 26 participants per group were required to be able to detect a difference between the groups at a 0.05 significance level with a power of 0.8.

## Results

### Group differences pre-training

Since groups were gender and age matched, no group differences were found with regard to age, gender or hand preference (Table 1). Also no differences between groups on weight, height or BMI were found (p = 0.35, 0.95, 0.40, respectively).

**Table 1:** Baseline characteristics of the participant groups.

Variables	TD Mean (SD) or n (%)	DCD Mean (SD) or n (%)
**Age (years)**	7.56 (1.02)	7.67 (1.02)
**Gender**		
**Male**	28 (51.9)	29 (50.9)
**Female**	26 (48.1)	28 (49.1)
**Hand Preference**		
**Right**	52 (96.3)	53 (93.0)
**Left**	2 (3.7)	4 (7.0)
**Body composition**		
**Height (m)**	1.24 (0.05)	1.21 (0.05)
**Weight (kg)**	26.9 (5.7)	28.3 (9.4)
**BMI (kg/m^2^)**	16.8 (3.1)	16.0 (2.7)

### Group differences pre-training on standardized tests

Since the children were selected based on their MABC-2 scores, large differences were shown for the MABC-2 Total Standard Score (mean TSS: TD 10.7 (2.1), DCD 3.77(1.3); (*t* (109) = 20.8, *p* < .0001). For means on pretests for all standardized tests see Table 2. Children with DCD scored worse on the sprint tests, running and agility, and balance. Additionally they performed worse on most of the functional strength tasks. No group differences emerged on the ‘lifting a box’ item.

**Table 2:** Means (SD) for the TD (n = 54) and DCD (n = 57) group and statistics of the independent t-test for the comparison at baseline.

Variables	Mean (SD) TD	Mean (SD) DCD	t(df = 109)	P-value	Cohen’s d
**MABC-2 Total score**	10.7 (2.1)	3.8 (1.3)	20.8	0.0001	4.06
**MABC-2 Subscore Manual dexterity**	10.9 (3.1)	5.8 (2.1)	15.5	0.0001	1.96
**MABC-2 Subscore Aiming & Catching**	9.8 (1.9)	6.0 (2.8)	8.3	0.0001	2.0
**MABC-2 Subscore Balance**	10.5. (2.65)	4.7 (2.0)	13.2	0.0001	2.51
**MABC-2 Balance AB3**	22.9 (6.7)	15.18 (7.8)	5.2	0.0001	1.06
**Long jump (cm)**	108 (21.0)	86.1 (19.9)	5.8	0.0001	1.07
**Mean Lat step (#)**	35.5 (5.8)	32.8 (6.9)	2.1	0.034	0.4
**Sit to stand (#)**	25.4 (6.3)	24.2 (7.3)	0.9	0.035	0.2
**Stairs (#)**	68.4 (11.5)	58.4 (10.5)	4.8	0.0001	0.9
**Lifting a box(#)**	17.2 (4.2)	15.8 (3.6)	1.9	0.48	0.4
**Sprint strait (s)**	23.8 (2.5)	25.9 (3.8)	-3.5	0.0001	0.7
**Sprint slalom (s)**	29.5 (4.3)	32.5 (7.3)	-2.6	0.0001	0.5
**BOT balance (Scale)**	17.7 (3.6)	13.9 (3.3)	5.7	0.0001	1.1
**BOT agility (Scale)**	20.1 (2.8)	16.6 (3.2)	5.8	0.0001	1.2

### Effects of training

The effect of training is summarized in Table 3. Overall, it can be seen that after the training children scored better on all activities tested, although the degree of improvement varied. It is also clear from the statistics that there was no main effect of protocol. Outcome of the paired sample t-test comparing pre and post measures for the DCD and TD group separately, is presented in Table 4. Results will be discussed in detail below.

**Table 3:** Repeated measure ANOVA Statistics (F, p and eta–values) for the main effects for the 3 factors (Group: TD/DCD; Training: pre- post and Protocol: Repetitive/Variable).

	Group			Training			Protocol		
	F	P	η2	F	P	η2	F	P	η2
**MABC-2 Total score** ^a^	220.67	0.0001	0.673	116.25	0.0001	0.601	0.027	0.871	0.0001
**MABC-2 Manual dexterity**	41.894	0.001	0.281	16.276	0.0001	0.132	0.060	0.808	0.001
**MABC-2 Aiming & Catching** ^b^	12.53	0.001	0.105	66.76	0.0001	0.384	0.317	0.575	0.003
**MABC-2 Balance** ^a^	64.228	0.0001	0.375	91.827	0.0001	0.462	0.001	0.977	0.0001
**MABC-2 Balance AB3**	28.99	0.0001	0.21	25.74	0.0001	0.194	0.329	0.33	0.009
**Long jump (cm)** ^a^	26.72	0.0001	0.203	35.632	0.0001	0.253	1.994	0.161	0.019
**Mean Lat step (**#**)**	4.45	0.037	0.041	85.811	0.0001	0.45	0.219	0.641	0.002
**Sit to stand (**#**)**	4.37	0.039	0.040	91.021	0.0001	0.464	1.370	0.245	0.013
**Stairs (**#**)**	13.70	0.0001	0.115	52.586	0.0001	0.334	0.130	0.911	0.000
**Lifting a box(**#**)**	6.85	0.01	0.061	119.366	0.0001	0.532	0.107	0.745	0.001
**Sprint strait (**s**)**	19.52	0.0001	0.157	10.149	0.002	0.088	0.056	0.813	0.001
**Sprint slalom (**s**)**	18.18	0.0001	0.148	67.221	0.0001	0.39	0.198	0.657	0.002
**BOT balance (Scale)** ^b^	33.90	0.0001	0.241	41.171	0.0001	0.278	0.306	0.581	0.003
**BOT agility (Scale)**	38.13	0.0001	0.265	35.35	0.0001	0.25	1.137	0.289	0.011

^a^ Interaction Participant group by Training

^b^ Interaction Participant group by Training x Protocol.

Interaction effects are not shown in the tables but described in the text or shown in graphs.

^#^ number

^s^ seconds

**Table 4:** Means (SD) and p-values of the paired sample t-test for the pre and post intervention outcomes for the TD and DCD group separately.

	TD pre	TD post	p	DCD pre	DCD post	p
**MABC-2 Total score (**SS**)**	10.7 (2.1)	12.1 (3.1)	0.004	3.8 (1.3)	8.6 (2.7)	0.0001
**MABC-2 Manual dexterity (**SS**)**	10.9 (3.1)	11.6 (3.2)	0.15	5.8 (2.1)	8.8 (2.3)	0.0001
**MABC-2 Aiming and Catching (**SS**)**	9.8 (1.9)	11.1 (2.8)	0.001	6.0 (2.8)	9.1 (3.2)	0.0001
**MABC-2 Subscore Balance (**SS**)**	10.5 (2.6)	11.5 (3.7)	0.094	4.7 (2,0)	8.8(3.5)	0.0001
**MABC-2 Balance AB3 (Sum score)**	22.9 (6.7)	26.0 (7.7)	0.001	15.18 (7.8)	19.25 (8.9)	0.001
**Long jump (cm)**	108 (21.0)	116.2 (25.3)	0.002	86.1 (19.9)	100.9 (19.1)	0.0001
**Lateral step (**#**)**	35.5 (5.8)	43.6 (7.4)	0.0001	32.8 (6.9)	42.5 (7.2)	0.0001
**Sit to stand (**#**)**	25.4 (6.3)	34.9 (7.6)	0.0001	24.2 (7.3)	32.3 (5.5)	0.0001
**Stairs (**#**)**	68.4 (11.5)	75.5 (16.5)	0.0001	58.4 (10.5)	69.4 (12.6)	0.0001
**Lifting a box (**#**)**	17.2 (4.2)	23.7 (6.4)	0.0001	15.8 (3.6)	20.8 (5.7)	0.0001
**Sprint strait (**s**)**	23.8 (2.5)	22.6 (2.2)	0.002	25.9 (3.8)	25.1 (3.1)	0.075
**Sprint slalom (**s**)**	29.5 (4.3)	24.0 (3.3)	0.0001	32.5 (7.3)	26.4 (3.6)	0.0001
**BOT balance (Scale)**	17.7 (3.6)	19.6 (3.4)	0.0001	13.9 (3.3)	16.7 (4.1)	0.0001
**BOT agility (Scale)**	20.1 (28)	21.8 (3.7)	0.0001	16.6 (3.2)	18.18 (4.2)	0.0001

^SS^; standard score

^#^; number

^s^; seconds

#### MABC-2

Results revealed a large effect of training (*F* (1,107) = 116.250, *p* <0.0001, *η*^2^ = 0.52) on the TSS. Against our hypothesis, the transfer of the active computer training to total MABC-2 scores was not influenced by the protocol (*p* = 0.87, *η*^2^<0.0001). However change on the MABC-2 total score was related to participant group (Interaction Training x Participant group *F* (1,107) = 35.49 *p* <0.0001, *η*^2^ = 0.249). The DCD groups improved more than the TD groups, both on total score and cluster balance. Nevertheless the change in both groups was significant on the TSS, with very large effect size for the DCD group (TD *F* (1,52) = 9.04 *p* <0.004, *η*^2^ = 0.148; DCD *F*(1,55) = 190.73 *p* <0.0001, *η*^2^ = 0.776).

Since the games played during training were predominately focused on balance and agility we included different test items to test transfer.

On the cluster of the MABC-2 balance items, large effect of training was found (*F*(1,107) = 48.12, *p* <0.0001, *η*^2^ = 0.31) but no interaction with protocol was revealed. An interaction with participant group was found (Interaction Training x Participant group *F*(1,107) = 18.71 *p* <0.0001, *η*^2^ = 0.149). On the balance cluster the improvement was only significant in the DCD groups and not for TD (Cluster Balance: TD *F*(1,52) = 2.88 *p* <0.094, *η*^2^ = 0.053; DCD *F*(1,55) = 73.95 *p* <0.0001, *η*^2^ = 0.573).

When we analyzed the MABC-2 balance tasks of the items of age band 3 again an effect of the training was shown (Balance AB3 *F*(1,107) = 28.62 *p* <0.0011, *η*^2^ =) 0.21 and no interaction with protocol emerged (*p* = 0.27) nor with participant group (*p* = 0.88). Post hoc analysis showed that both groups improved significantly on the more difficult items of the Balance items of the higher age band (Balance AB3: TD *F*(1,53) = 14.88 *p* <0.0001, *η*^2^ = 0.22; DCD *F*(1,55) = 13.75 *p* <0.0001, *η*^2^ = 0.20). This is a clear indication that the interaction found on the balance tasks of the appropriate age band was due to a ceiling effect for the TD group.

**Fig 2:**
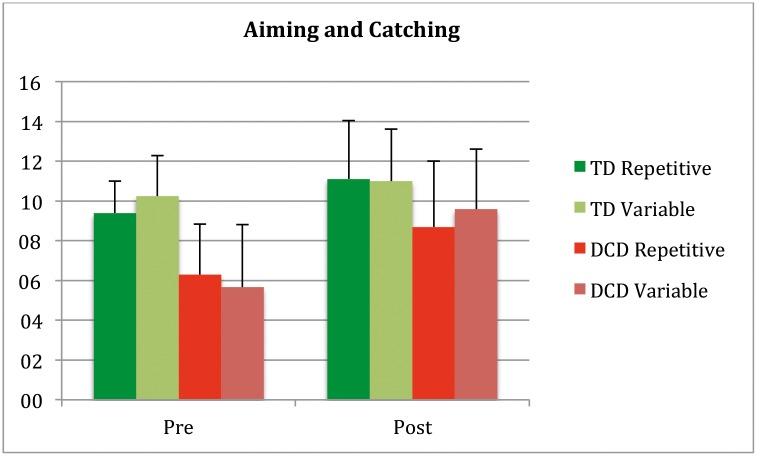
MABC aiming and catching pre and post.

Next, the component scores aiming and catching and manual dexterity of the MABC-2, with less common motor elements to the exergames were examined. For aiming and catching, main effects were significant (Training *F*(1,107) = 67.27 *p* = 0.0001, *η*^2^ = 0.39; Participant group *F*(1,107) = 42.55 *p* = 0.0001, *η*^2^ = 0.29) and the interaction of training by Participant Group (*F* (1,107) = 12.83 *p* = 0.001, *η*^2^ = 0.11) was significant and also Training by Group by Protocol (*F* (1,107) = 5.73 *p* = 0.018, *η*^2^ = 0.05) (Fig 2).

Post hoc showed no difference between participant groups in the repetitive protocol (*p* = 0.47), they both improved (TD *t*(25) = -3.32, *p* < .003, DCD *t*(28) = -3.66, *p* < .001), while in the variable protocol there was a difference in the effect of protocol (*F*(1,54) = 22.39 *p* = 0.0001, *η*^2^ = 0.29). The TD group did not improve in the variable training (*p* = 0.12) and the DCD group did (*t*(27) = −8.15, *p* < .0001).

Although no tasks requiring individual finger movements were trained, the children improved on the cluster score manual dexterity (*F* (1,107) = 41.97 *p* = 0.0001, *η*^2^ = 0.28). No main effect of protocol was found nor a training by protocol interaction. The main effect for participant group was significant (*F*(1,107) = 89.047 *p* = 0.0001, *η*^2^ = 0.45) as was the interaction training by participant group (*F* (1,107) = 16.36 *p* = 0.0001, *η*^2^ = 0.13). Post hoc showed that the TD group did not improve in manual dexterity while the DCD group did (TD *t* (53) = -1,456, *p* = 0.15; DCD (*t* (56) = -8.706, *p* < .0001).

#### BOT-2 component balance and running speed & agility

**Fig 3:**
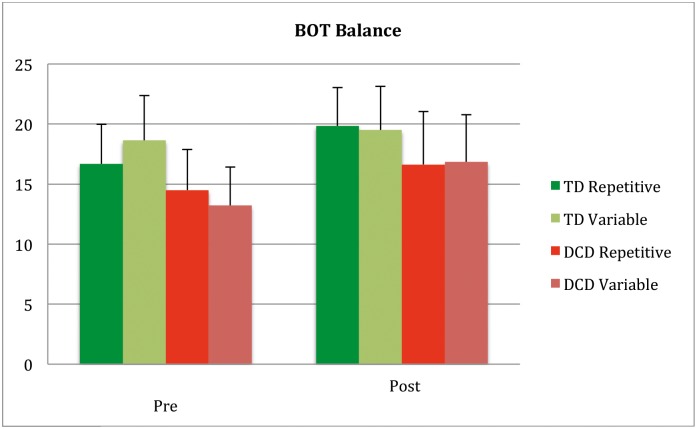
Depicting BOT-2 Balance scale scores pre post training for participant groups and protocol.

A comparable interaction pattern emerged when analyzing the Balance subscore of the BOT-2. A main effect of training was found (*F* (1,107) = 41, 17 *p* <0.0001, *η*^2^ = 0.278). Here also an interaction with participant group and protocol was found (Interaction Training x Participant group X Protocol *F* (1,107) = 7.25 *p* <0.008, *η*^2^ = 0.063) (Fig 3).

On the balance cluster the change was significant in both protocols in the DCD groups but for the TD there was an interaction with protocol *(F*(1,52) = 4.28 *p* <0.044, *η*^2^ = 0.078). The TD group improved on the BOT-Balance after the repetitive training (*t*(25) = −4.00, *p* < .0001) but not after variable training (*p* = 0.28).

A main effect of training (*F* (1,107) = 1,23 *p* = 0.27, *η*^2^ = 0.011) was found on the Running and Agility subscore of the BOT-2 but no interactions (Interaction Training x Participant group X Protocol *F* (1, 107) = 3,39 *p* 0.068, *η*^2^ = 0.031).

#### FSM

Based on earlier research [2] we also included assessment of daily activities that require functional strength (Long jump, Lateral step up, Sit to stand, Running stairs, Lifting a box). Training had a significant effect on all 5 functional strength test items (see Table 3). Importantly no interaction with protocol was found for any of these variables. For long jump an interaction of training with participant group was found (*F* (1,105) = 4.21 *p* = 0.043, *η*^2^ = 0.039) indicating that the DCD group improved more than the TD children. For the other FSM activities, no interactions with participant group emerged.

#### Sprinting (5x10 meter straight and slalom tests)

For the anaerobic sprint tests a main effect for training was found (Table 3). The effect sizes for the slalom were larger (*η*^2^ = 0.389) than for the strait sprint (*η*^2^ = 0.088), which fits with our prediction that agility can be improved by playing active computer games and not so much anaerobic fitness. No significant interactions with participant group or protocol were found.

To summarize, it can be concluded that both protocols lead to training effects on activities that require functional strength and tasks that require agility for both TD children and children with DCD. TD children are gaining less on the long jump or far transfer tasks like manual dexterity and aiming and catching.

## Discussion

To promote active computer games for skill learning in children with DCD, it must enable the child to transfer learning from the virtual environment to his or her real world environment. In this study, we tried to answer three questions about transfer of motor skills in a population of children with DCD and their typically developing peers. First, we tested if participating in 5-week exergaming training leads to improvements in every day motor skills. Second, we tested if variable practice would yield greater improvements in motor skills with common elements and less common elements than repetitive practice. Thirdly, we sought to establish if improvements observed would differ between TD and DCD children.

In the present study, we used the Nintendo Wii Fit, an active motion steered computer system, which highly motivates children [2–5,53], offers a high degree of time on task and gives augmented feedback during and after each task. Although the games were selected on the presence of whole body movements, half of the children had, to a certain extent, some control over their training sessions, depending on which group they were in. In this study, we have demonstrated that playing active video games provides positive transfer effects on balance tasks (hopping), running and agility tasks and functional activities such as long jump, sit-to-stand, and stair climbing. Similarly, we have shown when dynamic balance tasks are trained with exergames, it also leads to some improvements in ball skills. Importantly, we showed that transfer was not better for participants in the variable practice schedule compared to participants in the repetitive protocols. Although our findings were unexpected, we are not the only ones who found that variable practice does not always lead to better transfer. In a 2002 review, Wulf and Shea questioned the generalizability of results from studies using simple laboratory tasks to the learning of complex motor skills, especially in relatively young children. In cases of high attention, memory and motor demands, they argued that indiscriminate training may *overload* the system and thus disrupt the potential benefits of random practice. Blocked or repetitive training might be more effective, especially early in the learning process. Obviously, the novelty factor reduces faster during the repetitive protocol compared to the variable protocol, but the similar transfer and scores on enjoyment suggests that there was no effect (yet) after five weeks. A certain level of experience may be required for variable training to become more effective than blocked or repetitive training. Also the rules for transfer of learning may be different between children and adults, or for experts and novices in more complex tasks. [54–58].

### Type of practice and transfer to skills with identical elements

The VR environment provides an enabling environment for training motor skills in healthy subjects and those with conditions affecting motor function. This is because it provides frequent augmented feedback in the form of visual and auditory signals. Augmented visual feedback helps children to refine movement patterns to produce coordinated movements [59]. In our study, we found that both TD and DCD children improved in balance skills as well as in tasks such as slalom running, sit-to stand and lifting a box. This transfer effect was similar regardless of training protocol. This finding contradicts what has been previously reported in the literature [60]. It has been suggested that variable practice offers greater transfer benefits in children and adults. In practice variability studies that have been conducted with the Nintendo Wii, participants showed great gains in transfer when tested on agility drills [2,3]. However, in the present study, both variable and repetitive practice yielded similar transfer effects in DCD and TD children. This suggests that amount of transfer of skills from exergaming to the physical world is not dependent on the type of practice structure.

According to Schmidt (1987)[15], a well-learned skill is adaptable and could easily transfer to novel contexts. This observation could be explained by the identical elements theory [16]. Participants played games that required weight shifting, anticipatory control, reactive control, trunk and lower extremity control. The movement patterns that children employed in these game situations were similar to the majority of the real life skills such as sprinting and avoiding other children while running on a playground during a game of tag or climbing a flight of stairs. Due to the increased number of common elements between trained skills and those evaluated on motor tests, children found it effortless to generalize balance skills they acquired in game settings to their natural environment.

### Type of practice and transfer to skills with less identical elements

From the findings presented in this paper, it is clear that children in the TD and DCD group also increased performance in skills that were not specifically trained. This result indicates that playing exergames could lead to motor skills transfer from practiced skills (balance and agility) to unpracticed skills (object control). Although the effect size of the change in aiming and catching (0.38) is smaller than for balance (0.46), this seems partly at odds with the theories of training specificity and identical elements. While the MABC-2 items aiming, and catching are very static, anticipation to moving objects and postural adjustments to fast moving body parts were trained in both training protocols. The indissoluble relation between balance and postural control may have led to improvements in fast postural changes and response time, which are key requirements for aiming and catching. Moreover, an enhanced ability to extract goal-relevant task characteristics would allow players to better predict upcoming events and to allocate perceptual and cognitive resources in a manner to take advantage of those predictions. To a certain extent, it is possible that the children improved in anticipatory control, sensory information processing capacities (both visual and auditory), and attention focus resulting in transfer effect even across untrained skills. The visual pursuit of the avatar during the games, which also requires sustained visual attention, could have led to small beneficial effects on manual dexterity tasks in children with DCD. This interpretation is supported by the transfer-appropriate processing theory [17], which explains that, regardless of the type of practice or group allocation, participants showed positive far transfer albeit to a lesser extent to dissimilar skills (Effect size 0.46 for Balance, 0.13 for Manual Dexterity).

### Differences in transfer between participant groups

Contrary to our expectations, we observed no differences in skill transfer between the DCD and TD children. This means that motor coordination deficits have little or no influence on transfer of the skills tested. Given that transfer effects did not differ significantly between groups, it can be argued that the mechanisms underlying this process in a VR environment is similar in children with and without DCD. This suggests that an active video game does not only allow children with DCD to learn new skills but also promotes skill transfer to both trained and untrained tasks. This is an important finding given that these children struggle to transfer skills acquired in traditional rehabilitation clinic to real world contexts.

The three two-way interactions (Training by Group) found in our study, are clearly the result of ceiling effects of the measures in the TD children (both BOT and MABC balance items, and Ball skills subtests of the MABC had maximum scores for most of the TD children). The fact that TD children did not improve on the BOT balance items and on the component balance of the MABC-2 when tested on the appropriate age band, but did improve when tested on more difficult items of the higher age band of the MABC, stresses that intervention research needs to include tasks that have sufficient room for improvement given the level of proficiency.

This ceiling effect is probably also the reason of the 2 three-way interactions found (Training by Group by Protocol). The most likely explanation is, the slightly (non-significant) lower initial level of the MABC-2 aiming and catching and BOT balance of the repetitive Typically Developing group, leaving a little space for improvement in that group, while maximum scores were already reached for the Variable group at pretest. (Figs 1, 2 and 3).

### Strengths and limitations

One could argue that although children assigned to repetitive practice played only one game, the structure was not really repetitive practice. Critically, it is evident that each trial of the ski slalom game presented slightly different challenge to the player. For example, in the course of the game, the children were bombarded with multiple forms of sensory information that was meant to assist them become better players on subsequent trials. Although the ski game has a fixed layout and does not vary its spatial configuration, the amplitude and the temporal aspects of the movement made by the child varied over training session as children improved. This might have introduced some form of variability in the game itself. The child will implicitly learn to minimize the motion for maximum results (earning more points) and do so in a more anticipatory way, optimizing the trajectory if they play the game for a prolonged period of time.

Another limitation is the duration of the study (5 weeks). As reported earlier, the outcome may have been different if we doubled the duration of the training. Not only would the repetitive protocol become boring leading to a probable difference in degree of fun, it would be interesting to see whether learning levels would be still different in the TD compared to the DCD group. It also would be interesting to study whether children would still benefit from improved skills after a longer period of time and a follow-up study is recommended.

Strength of the study is that we had large groups (>25) that were randomly assigned to the conditions. All children had the same time on task and the evaluators were blinded to the pretests results, thus a design that excluded many possible biases. Lastly, we used a large range of transfer tasks to be able to conclude that skill transfer from virtual environment to real world context occurred.

## Conclusions

Exergaming leads to improvements in every day skills. The skills acquired in a VR environment can transfer to real life contexts, which makes exergames effective training tools for children with fewer opportunities to play (e.g. no safe play areas, constrained to in-door activities) and with neurodevelopmental disorders like DCD. The two protocols used in this study yielded comparable effects, with larger effect sizes in motor skills with common elements than in skills with less common elements. Improvements in motor skills did not differ between children with DCD and their typically developing peers, albeit on a different level. Importantly, the degree of skill transfer was not influenced by the amount of variation in the games played during a 5 weeks training period.

## Supporting information

S1 Appendix (PDF)
