# Supplementary material for: Learning better by repetition or variation? Is transfer at odds with task specific training?
Source: PLoS One. 2017 Mar 23;12(3):e0174214. doi: 10.1371/journal.pone.0174214 (PMC5363924; doi:10.1371/journal.pone.0174214)
Supplement: S1 Appendix — (PDF) [file pone.0174214.s001.pdf]

Appendix

All games selected aim at functional strength, anaerobic fitness, balance skills and agility

1. Heading
Steer the Mii character by shifting your weight left and right on the balance board. Goal is to try and head the incoming soccer balls but avoid heading the incoming pandabear heads & soccer shoes.

2. Table Tilt
Steer the floating table by shifting your weight forwards, backwards, left & right. Goal is to steer the marbles down through the holes.

3. Ski-jump
Steer the Mii character down the ski-jump by leaning forward with bended knees holding still and jump by extending the legs swiftly right before the red line. Goal is to keep the Mii character balanced during the flight in order to jump as far as possible.

4. Balance Bubble
Steer the bubble with the Mii character inside by shifting your weight forwards, backwards, left & right. Goal is to steer the bubble down the river without hitting the sides or varying obstacles along the way.

5. Pinguïn slide
Tilt the iceberg by leaning left and right on the balance board. This way the penguin slides left and right. Goal is to let the penguin catch as many fish as possible. Each fish is worth a specific amount of points. For extra points the player needs to catch the biggest fish by shifting their weight swiftly to the opposite direction when the penguin is at the edge of the iceberg.

6. Snowboard slalom
The balance board needs to be turned 90 degrees with the left side of the balance board pointing at the television screen. Steer the Mii character down the slope by leaning backwards and forwards to steer and sideways to speed up or slow down. Goal is to pass between the gates as fast as possible.

7. Rhythm Kungfu
For this game the player needs to have the remote control in one hand and a nunchuck control in the other hand. The player needs to copy the Kungfu movements of the Mii characters one second after they have demonstrated the rhythmic variable moves of the arm(s) and leg(s) twice. Timing of the movement is essential to get the high score.

8. Obstacle course
Steer the Mii character through the obstacle course by lifting your feet slightly of the balance board in order to walk and extending the legs swiftly to jump. This way the Mii walks over a (moving) path or jumps over a depth or an obstacle. The goal is to get through the obstacle course as fast as possible avoiding the varying obstacles and reach higher levels.

9. Skateboarding
The balance board needs to be turned 90 degrees with the left side of the balance board pointing at the television screen. Steer the Mii character over the green lights located on variable obstacles to get points. Steer by leaning forwards and backwards, speed up by stepping off the balance board with one foot and jump by extending your legs swiftly. Goal is to get a certain amount of points within a limited amount of time to reach the next round/level.

10. Perfect 10
Hit the right numbers adding up to 10 by moving your hips forwards, backwards, left or right.
